# Verification policies in Croatian medical biochemistry laboratories: a survey of the practice

**DOI:** 10.11613/BM.2022.020703

**Published:** 2022-04-15

**Authors:** Lara Milevoj Kopčinović, Gordana Juričić, Adriana Bokulić, Ines Vukasović, Ivana Ćelap, Helena Čičak, Marija Kocijančić, Marija Miloš, Mila Lovrić, Marija Siter Kuprešanin, Snježana Hrabrić Vlah, Manuela Miletić

**Affiliations:** 1 Working group for method verification and validation of the Croatian Society of Medical Biochemistry and Laboratory Medicine and Croatian Chamber of Medical Biochemists, Zagreb, Croatia; 2 Department of Clinical Chemistry, Sestre milosrdnice University Hospital Center, Zagreb, Croatia; 3 Department of Laboratory Diagnostics, General hospital Pula, Pula, Croatia; 4 Laboratory of Endocrinology, Department of Oncology and Nuclear Medicine, Sestre milosrdnice University Hospital Center, Zagreb, Croatia; 5 School of Medicine, Catholic University of Croatia, Zagreb, Croatia; 6 Department of Medical Laboratory Diagnostics, University Hospital “Sveti Duh”, Zagreb, Croatia; 7 Department of Laboratory Medicine, Central Laboratory, University Clinic Halle, Halle (Saale), Germany; 8 Department of Laboratory Diagnostics, University Hospital Centre Zagreb, Zagreb, Croatia; 9 Faculty of Pharmacy, University of Mostar, Mostar, Bosnia and Herzegovina; 10 Faculty of Pharmacy and Biochemistry, University of Zagreb, Zagreb, Croatia; 11 School of Medicine, University of Zagreb, Zagreb, Croatia; 12 Clinical Department of Laboratory Diagnostics, Clinical Hospital Center Rijeka, Rijeka, Croatia; 13 Laboratory of Medical Biochemistry, Zagreb County Health Center, Velika Gorica, Croatia

**Keywords:** accreditation, examination procedures, verification, survey

## Abstract

**Introduction:**

The aim of this study was to screen practices used in verification procedures for methods/analysers among medical biochemistry laboratories (MBLs) in Croatia. We hypothesized that these procedures differ widely from laboratory to laboratory and wanted to gather specific data on steps used in the verification workflow.

**Materials and methods:**

In order to obtain data, an online survey was conducted. The survey, divided in two sections, contained 29 questions and statements addressing general characteristics and specific steps of the verification workflow of each individual MBL. The survey was disseminated among managers of all MBLs in Croatia.

**Results:**

A total of 108/196 (55%) laboratories participated in the survey. Forty nine MBLs were excluded from the second part of the survey: 14 have not implemented verification procedures, and 35 MBLs due to the absence of answers. The most relevant results of the second part of the survey showed that: 18/59 (0.31) of the responding MBLs have difficulties when defining acceptance criteria, 27/59 (0.46) used the Clinical and Laboratory Standards Institute protocol for precision estimation; the majority of MBLs used a median of 20 samples for method/analyser comparisons and estimated bias using internal quality control samples; reference intervals provided by external sources are mainly adopted; 60% of MBLs do not include linearity verification in their protocol and do not use the national document for the estimation of measurement uncertainty.

**Conclusions:**

Heterogeneous verification protocols are routinely utilized across Croatian MBLs which clearly confirms that a national document might help in the harmonization of verification procedures.

## Introduction

The verification process is an important precondition in the pursuit to ensure reliable, high quality laboratory test results and ultimately increase patient safety. The term verification encompasses the provision of objective evidence that a measurement procedure/measuring system meets the manufacturer´s performance requirements (*1*). As an essential requirement, it is embedded in the International Standard ISO 15189 intended specifically to guide the management of quality systems in medical laboratories (*2*, *3*).

According to the IVD Medical Device Directive 98/79/EC, manufacturers of *in vitro* medical devices should perform the validation of measurement methods/systems marketed in Europe (*4*). In order to ensure patient safety and compliance with ISO 15189 accreditation requirements, medical laboratories have the responsibility to independently confirm performance properties stated by the manufacturer before implementation of an examination procedure into routine practice (*5*). Verification of performance properties comprises an appropriate design and execution of a series of experiments in order to ensure that the measurement procedure or measuring system meets the manufacturer´s claims and confirms that it is fit for the intended use. However, the optimal extent of the verification procedure (*i.e.* a detailed description of the procedure with regard to different measurement methods/measuring systems) applicable for all laboratories, and the mathematical basis of calculations used are not specified in the ISO 15189 standard (*3*, *6*). The absence of a comprehensive guideline that could assist medical laboratories in designing and performing each step of the verification process results in widely different definitions of local policies for verification purposes. Since locally tailored procedures mainly rely on individual interpretation of available documents describing the verification procedural workflow, they are often inconsistent and not balanced in terms of heterogeneity of method types, local technical capabilities, increased workload, costs and risks.

Harmonization of verification procedures might be accomplished by compiling national documents, taking into account specific aspects and demands of local laboratories while equilibrating them within the mandatory requirements of the ISO 15189 standard (*7*). Considering that these issues have not been adequately addressed in Croatia, the Croatian Society of Medical Biochemistry and Laboratory Medicine (CSMBLM) and Croatian Chamber of Medical Biochemists (CCMB) formed a Working group for method verification and validation (WG VV). The specific goals of the WG VV can be summarized as follows: 1) to identify current policies and practices used in performing verification experiments among medical biochemistry laboratories (MBLs) in Croatia, 2) to identify main problems related to the performance of verification, and 3) to propose national recommendations that would facilitate and guide Croatian MBLs while performing method verification. The ultimate goal is to harmonize verification procedures on the national level while taking into account the difficulties encountered in small MBLs as well as the capacities of large MBLs. Accordingly, we hypothesized that verification procedures performed in Croatian MBLs differ widely from laboratory to laboratory. Thus, the aim of this survey was to accomplish the first goal of the WG VV, *i.e.* to gather specific data on steps used in the verification workflow and problems occurring while performing method verification in Croatian MBLs.

## Materials and methods

In order to obtain data on verification procedures used in Croatia, two rounds of the survey were conducted using the online survey platform SurveyMonkey (SurveyMonkey Inc., Palo Alto, USA). First, a pilot survey was sent to 15 randomly chosen managers of MBLs belonging to different health care settings. The questionnaire contained 16 questions with predefined answers and allowed comments input. This pilot survey aimed to identify the appropriateness of the questions asked. Based on these first results, the members of WG VV prepared a second questionnaire intended for the second round of the survey which contained 29 questions/statements. The second survey was disseminated among managers of all MBLs in Croatia (N = 196) during February 2019. The managers were asked to fill in or select one or more of the proposed responses.

The second survey was divided in two sections. The first section included questions/statements on the type of laboratory (*i.e.* health care setting), accreditation status and implementation of verification procedures. The second section included detailed questions/statements related to the specific steps of the verification workflow performed in each individual MBL which implemented verification policies in routine practice.

### Statistical analysis

Results are presented as numbers and percentages (when N ≥ 100) or numbers and proportions (when N < 100).

## Results

A total of 108 laboratories participated in the survey, which represents 55% of MBLs in Croatia. The general characteristics of the participating MBLs are presented in Table 1. Most of the surveyed MBLs (81%) had been supervised by the CCMB, which mandates the fulfilment of a series of standards adopted from the ISO 15189. However, to date, only 10 (9%) of surveyed MBLs are accredited according to the ISO 15189 standard. In the last ten years, the majority of the participating MBLs declared to have implemented into routine practice one of the following: 1) a new analytical system (104/108, 96%); 2) a new method for an existing examination procedure (66/108, 61%); 3) a newly implemented examination procedure (76/108, 70%); 4) a new generation assay (63/108, 58%); 5) an assay of a different manufacturer (60/108, 56%). Only one MBL declared none of the above. Fourteen MBLs (13%) stated not to have implemented verification procedures in their routine practice, and were not surveyed in the second section. The main reasons for not implementing verification procedures, as stated by the participating MBLs, were organizational (due to lack of staff or time), absence of a document harmonizing verification procedures, lack of economic resources and lack of availability of the necessary data from the manufacturer.

**Table 1:** Characteristics of the participating Croatian medical biochemistry laboratories

**Laboratories by health care setting (N = 108)**	**N (%)**
Primary health care	57 (53)
Private health care	13 (12)
Secondary and tertiary health care	38 (35)
**Laboratories by accreditation status**	
Accredited	10 (9)
Accreditation in process	8 (8)
Not accredited	90 (83)
**Laboratories supervised by the CCMB**	
Yes	87 (81)
No	20 (18)
Planned	1 (1)
**Laboratories implementing verification procedures**	
Yes	94 (87)
No	14 (13)
Primary health care laboratories comprised those associated with primary care physicians. Secondary and tertiary health care laboratories comprised laboratories located in special hospitals, general hospitals, clinics, clinical hospitals and clinical hospital centres. CCMB – Croatian Chamber of Medical Biochemists.	

One third (35/94) of the MBLs declaring to implement verification procedures in the first part of the survey were excluded from the second part of the survey due to absence of answers (individual or all). The results obtained from the remaining laboratories, participating in the second part of the survey related to minimal verification requirements, are summarized in Table 2.

**Table 2:** Procedures pertaining to verification workflow used by Croatian medical biochemistry laboratories – minimal performance characteristics

		**Answers (N = 59)**		
**Question/statement**		**Yes** **(N, proportion)**	**No** **(N, proportion)**	**Sometimes** **(N, proportion)**
1. Verification of examination procedures is performed when implementing:*				
a new analytical system.		56 (0.95)	3 (0.05)	NA
a second analytical system identical to the one in routine use.		45 (0.76)	14 (0.24)	NA
a new generation assay.		36 (0.61)	23 (0.39)	NA
a new assay.		54 (0.92)	5 (0.08)	NA
a new sample type for an existing assay.		35 (0.59)	24 (0.41)	NA
a different method for an existing assay.		49 (0.83)	10 (0.17)	NA
2. Is defining acceptance criteria for verification results a problem in your laboratory?		18 (0.31)	41 (0.69)	NA
3. Acceptance criteria for verification results in your laboratory are defined according to:*				
clinical recommendations, depending on the intended use of the result.		37 (0.63)	22 (0.37)	NA
available biological variability data.		45 (0.76)	14 (0.24)	NA
the manufacturers claims.		42 (0.71)	17 (0.29)	NA
4. Is the verification study protocol for each examination documented?		35 (0.59)	24 (0.41)	NA
5. Does the verification study protocol contain explanations if some specifications of the examination were not tested?		16 (0.27)	43 (0.73)	NA
6. Precision is tested only with commercially available control materials.		38 (0.64)	21 (0.36)	NA
7. Precision is tested with patient’s samples if no commercial control material with values near the clinical decision making threshold is available.		32 (0.54)	27 (0.46)	NA
8. Is stability of the analyte verified for patient samples used during the time of data collection for precision and comparison studies?		11 (0.19)	48 (0.81)	NA
9. Does the verification study protocol include bias estimation?		35 (0.59)	14 (0.24)	10 (0.17)
10. How is bias estimation performed? *^†^				
Using method comparison data with patient samples.		19 (0.42)		
Using data from external quality assessment.		25 (0.56)		
Using internal quality control sample and its assigned value.		36 (0.80)	NA	NA
Using certified reference material.		4 (0.09)		
Using a different lot of calibrator and its “target value”.		7 (0.16)		
*Multiple answers allowed. ^†^Responding medical biochemistry laboratories N = 45. NA – not applicable.				

One third of the responding MBLs (18/59, 0.31) stated to have difficulties when defining acceptance criteria in order to assess verification results, despite the availability of a defined hierarchy of criteria (*8*). The most prominent problems linked to this crucial step of the verification process, as stated by the responding MBLs, were: a) availability of multiple sources of acceptance criteria which may be confusing; b) analytes lacking available biological criteria; and c) sometimes criteria cannot be achieved and thus minimal criteria should be defined.

For precision estimation, 27/59 (0.46) of MBLs used the Clinical and Laboratory Standards Institute (CLSI) EP15-A2 protocol - triplicate measurements of two concentrations daily for five days (*9*). Five MBLs perform triplicate measurements of three concentrations daily for five days, 4 MBLs one measurement of two concentrations daily for five days. Two MBLs perform 10 serial measurements of 3 concentration levels for five days while the remaining 21 MBLs declared to use 20 different combinations of replicates, concentration levels and days used in precision experimental design.

Two questions were related to the specifics of method comparison protocols used in the MBLs surveyed. A median of 20 samples (95% confidence interval (CI): 1-50) is used for method/analyser comparisons. The declared average time needed for comparison studies to be completed was 5 days (95% CI: 1-30). The majority of the responding MBLs (18/54, 0.33) use 20 samples in comparison experiments and need 1-30 days for sample/data collection. Fifteen MBLs declared to use 40 or more samples in comparison studies with a duration of 5-30 days. Eight MBLs use 30 samples through 5-20 days, and 6 MBLs use 10-15 samples through 3-20 days. Seven MBLs stated that they use 2 or less samples for method comparison protocol. As for the suitability of samples for comparison studies, 37/54 (0.69) MBLs can ensure samples with results covering the entire measuring range, while 13/54 (0.24) MBLs declared to collaborate with other MBLs in order to be able to obtain samples with concentrations covering the measuring range stated by the manufacturer. A half (27/54, 0.50) of the responding MBLs declared to implement dilution experiments (samples with high analyte concentrations, near the upper limit of the measuring range) in order to obtain comparison results.

Table 3 presents the results of the MBLs responding to the final part of the survey related to the verification of performance characteristics that might be included in a more extended (rigorous) approach. Out of 35 MBLs not verifying linearity of the measuring range, ten later stated to perform linearity verification but not for the entire range declared by the manufacturer. The most prominent problems encountered when performing a verification process, according to the participating MBLs, are: 1) financial issues associated with the experiment (41/53; 0.77); 2) collection of samples with concentrations covering the methods’ entire measuring range (38/53; 0.72); 3) lack of national guidelines detailing the steps of the verification process (37/53; 0.70); and 4) lack of a statistical software in order to ease the calculations (35/53; 0.66).

**Table 3:** Procedures pertaining to verification processes used by Croatian medical biochemistry laboratories – extended performance characteristics

		**Answers (N = 54)**	
**Question/statement**		**Yes** **(N, proportion)**	**No** **(N, proportion)**
1. Describe the RI verification procedure in your laboratory:*^†^			
It is performed for methods with no harmonized RI according to the CCMB.		25 (0.46)	29 (0.54)
It is performed for methods if manufacturer declared RI differ from those proposed by CCMB.		13 (0.24)	36 (0.66)
Manufacturer declared RI are implemented.		39 (0.72)	16 (0.30)
Available RI from literature data are implemented (if they are determined with the same method on the same population).		30 (0.55)	20 (0.37)
2. Do you estimate limit of blank (LOB)?		2 (0.04)	52 (0.96)
3. Do you estimate limit of detection (LOD)?		2 (0.04)	52 (0.96)
4. Do you estimate limit of quantification (LOQ)?		3 (0.06)	51 (0.94)
5. Do you evaluate linearity for the declared measuring range?		19 (0.35)	35 (0.64)
6. Do you perform diagnostic accuracy study where appropriate?		4 (0.07)	50 (0.93)
4. Do you estimate the accuracy of cut-off value where appropriate?		2 (0.04)	52 (0.96)
5. Do you estimate measurement uncertainty according to national guidelines?		21 (0.39)	33 (0.61)
6. If the verification results do not meet the acceptance criteria:*			
the method is considered not suitable for routine use.		20 (0.37)	34 (0.64)
the verification protocol is repeated using a new set of the same sample types (*e.g.* control samples of different lot or serial number, patient samples derived from different patients).		38 (0.72)	16 (0.30)
the verification protocol is repeated using different sample types (*e.g.* control samples instead of patient samples and *vice versa*)		30 (0.57)	24 (0.44)
*Multiple answers allowed. RI – reference interval. CCMB – Croatian chamber of medical biochemists. ^†^Two MBL declared not to perform verification of reference intervals (RI).			

## Discussion

This survey was conducted in order to screen current practices for verification of examination procedures among Croatian MBLs. Valuable information was gathered on specific aspects related to the verification procedure, and as expected, our main finding were the heterogeneous verification protocols used in MBLs across Croatia.

The majority of the responding MBLs were non-accredited primary health care MBLs which is expected given the high proportion of small MBLs due to the geographical properties of Croatia and the non-mandatory requirement for accreditation in Croatia. Most of the surveyed MBLs have been supervised by the CCMB, which means they have implemented a series of quality standards compiled according to ISO 15189. Thus, although not accredited, most MBLs in Croatia implemented a quality management system based on ISO 15189 which should include the verification of examination procedures. However, 13% of surveyed MBLs declared not to verify examination procedures which is in contrast to the accreditation encouragement in Croatia and favourable accreditation trend found in other members of the European Federation of Clinical Chemistry and Laboratory medicine (EFLM) (*10*). The most prominent reasons for not implementing verification into routine practice were organizational (technical) and financial; however, the lack of a national document harmonizing verification procedures was also emphasized.

Verification is predominantly performed by most MBLs when a new analytical system, a new assay or a different method for an existing assay is implemented. However, about one third of participating MBLs stated not to perform verification in situations when a new generation assay or a new sample type for an existing assay is implemented. In general, when modifying an examination procedure after it has been verified, the impact of such modification on the intended use should be carefully evaluated (preferably using a risk analysis approach) (*3*, *7*). If the modification is found to be relevant and potentially affecting the performance characteristics (as in the case of a new assay or sample type), a supplemental verification should be performed (*3*, *7*, *11*).

Furthermore, about 40% of participating MBLs in the second part of the survey declared not to document verification protocols for each examination procedure and only 27% of MBLs include an explanation if individual specifications are not examined. It has to be emphasized that a structured verification report (including the intended use of the examination procedure, description of the measurand, experimental plan including relevant performance characteristics, acceptance criteria, raw experimental data, concluding remarks and identity of the investigators) is an important element of the verification procedure which allows traceability of verification results to the requirements stated in the standard (*7*).

Acceptance criteria are defined based on the intended use of the examination procedure and should be defined prior to the verification experiment (*7*). The results of our survey show that the majority of the participating MBLs find defining acceptance criteria not an issue. Preferred sources of acceptance criteria are biological variation data, manufacturer’s claims and, finally, clinical recommendations. However, one third of the responding MBLs declare defining acceptance criteria an issue, and identify the availability of multiple sources of criteria and the lack of biological data as main problems. The majority of the responding MBLs find that measurement procedures which do not meet the predefined verification criteria are not suitable for implementation into routine practice; however they also tend to repeat the verification process by using either a new set of same samples or a new set of different samples. Each verification result that doesn’t meet the acceptance criteria should be observed through risk analysis. It is necessary to verify the probability, severity, and the meaning of the given result if the method is to be accepted for routine work. In terms of verification extent (*i.e.* which characteristics to verify) and specific ways how to perform verification (*i.e.* how to evaluate each performance characteristic) the ISO 15189 standard is merely a framework (*1*, *7*). The responsibility of determining the extent of verification and evaluating the results obtained are left to laboratory professionals of individual MBLs. This allows the design of verification experiments that meet the requirements without becoming too prescriptive, but at the same time causes great heterogeneity in terms of experiments performed, as demonstrated by our results. The majority of participating MBLs include minimal protocols when verifying an examination procedure: imprecision and bias. Their combination can be used to assess measurement uncertainty in order to provide information on the examination’s accuracy (or lack of it). Imprecision depends on local conditions and thus must be assessed experimentally (*3*, *7*). Participating MBLs stated that imprecision is preferably tested using commercially available control materials, and only in their absence using patients’ samples, although patient samples and pooled patient samples are the best available materials for imprecision studies (*9*). Furthermore, the design of local protocols for imprecision testing varies widely in participating MBLs potentially affecting the reliability of repeatability and within-laboratory imprecision estimates.

The majority of participating MBLs estimate bias preferably using internal quality control samples (and their assigned values), followed by using data from external quality assessment and method comparison studies using patient samples. The first approach is invalid for the assessment of bias, as is the approach of using calibrators of different lots, declared by 7 participating MBLs. Interestingly, four MBLs declared to use certified reference materials for estimating bias. For bias estimation, samples with known “true” concentrations of the measurand (*e.g.* reference standards, external quality survey materials, interlaboratory quality control programs) are recommended (*12*, *13*). However, due to its practicality, the most common approach for bias estimation is the comparison procedure using patient samples. It is important to keep in mind that this approach implies estimation of difference between the candidate and comparative measurement procedure and not the actual bias (*13*). Only 15 MBLs stated to follow the CLSI protocol for comparison studies using patient samples, *i.e.* a minimum of 40 samples. The majority of MBLs declared to perform a comparison procedure using less than 30 samples (some reaching as low as 2 samples) which is unacceptable since it might seriously affect the soundness of statistical analysis and jeopardize the confidence of the results obtained (*13*). Samples for comparison studies should be chosen carefully in order to cover the entire measuring range and to comply with measurand stability (*11*, *13*). If obtaining samples with concentrations covering the measuring range is not possible, participating MBLs declare to collaborate with other laboratories in order to fulfil this requirement. However, 81% of the participating laboratories stated not to verify the stability of the samples used for comparison studies which might unnecessarily introduce sample stability as a variable and compromise the results of the comparison study (*13*).

Although it is not realistic to expect that each laboratory will develop its own RI, the adequacy and usefulness of available RI from other sources should be verified in each individual laboratory (*14*). Croatian MBLs mainly adopt RI provided by three sources: the CCMB, manufacturers and/or those available from the literature (*15*). Accordingly, the practice about RI verification were different among laboratories. It is expected that the verification report contains a conclusion of the RI to be applied (*e.g.,* applicability in relation to the method and/or patient population). If MBLs want to verify manufacturers or literature’s data, the verification procedure is not a time consuming and costly task for a majority of analytes and may be performed with as few as 20 samples from reference individuals or using data from the method comparison study (*14*).

Detection capability estimates are important performance specifications for measurement procedures which need to reflect quantitative reliability in the low end of the measuring range. The decision on which estimate to verify (limit of blank (LOB), limit of detection (LOD) and/or limit of quantification (LOQ)), depends on the particular measurement procedure (*16*). For example, LOD and LOQ are important characteristics when extremely low amounts of the measurand have clinical significance (disease diagnosis and screening, presence or absence of substances, *etc.*). Unfortunately, our results showed that Croatian MBLs in general do not implement the estimation of LOB, LOD and LOQ in their verification protocols.

Linearity is usually provided by manufacturers and its inclusion in the verification protocol is not mandatory (*3*, *5*, *17*). About 60% of the responding MBLs declared not to include this key analytical characteristic in their verification protocol.

A qualitative test gives a binary result, *i.e.* positive/negative. Some qualitative tests produce a numerical response/ratio which is translated into a dichotomous result by comparison to the corresponding cut-off value. Usually, the manufacturers provide cut-off concentration values for different purposes (screening, diagnosis, disease management) but sometimes recommend to the laboratory the establishment of their own cut-off values (*5*, *18*, *19*). According to the responses related to the verification protocols for qualitative test, the majority of participating MBLs do not implement the estimation of cut-off values accuracy and evaluation of diagnostic performance characteristics in their verification protocols. These results are quite surprising and indicate the urgent need to address this issue with a comprehensive national document.

Measurement uncertainty depicts a values range where a true value of the measurand (or a quantifiable property of the analyte) could be expected with a given probability (*20*). It is easily included in the verification protocol since it can be estimated from data obtained during the verification experiment. Despite the proposed purpose of the national document for the estimation of measurement uncertainty, harmonization has not taken root in practice, since 60% of the responding MBLs declared as not using these national guidelines.

Our study has some limitations that need to be pointed out. Firstly, the response rate of our nationwide survey was 55%, which means that our results may not be representative of all Croatian MBLs. Furthermore, data from our participants were self-reported and thus could not be independently verified. Additionally, since this is the first survey investigating national verification procedures, we were unable to compare our results to similar studies. Thus, the obtained results were compared and discussed in relation to available recommendations mainly issued by the CLSI or EFLMs position papers.

In conclusion, heterogeneous verification protocols are routinely implemented in Croatian MBLs. This confirms that a national document on verification of examination procedures, tailored and optimized specifically for local demands and circumstances, might help not only in the harmonization of verification procedures but also facilitate the verification process in order to achieve that all MBLs in Croatia perform verification of examination procedures.
